# 772. Effect of Selective Digestive Decontamination Using Oral Colistin on HAI Rates and All-Cause Mortality Among Cardiovascular Surgery Patients - A Single Centre Experience from India

**DOI:** 10.1093/ofid/ofab466.969

**Published:** 2021-12-04

**Authors:** Sneha R, Arun Wilson, Anup R Warrier, Shilpa Prakash, Reima Elizabeth, Suresh Nair

**Affiliations:** 1 Aster medcity, Kochi, Kerala, India; 2 Aster Medcity, kochi, Kerala, India

## Abstract

**Background:**

Hospital acquired infections affect the morbidity and mortality of ICU patients considerably. Selective digestive decontamination (SDD) is defined as the prophylactic application of topical, non-absorbable antimicrobials in the oropharynx and stomach, with the goal of eradicating potentially pathogenic microorganisms but preserving the protective anaerobic microbiota. SDD has been applied in trials among critically ill patients and found to be effective in reducing HAI.

**Methods:**

This cohort study was conducted in our cardiothoracic vascular surgery ICU of a tertiary care hospital, where patients were given oral colistin syrup (100mg 6^th^ hourly for 5 days) in the immediate post op during the intervention period. We compared the clinical and microbiological outcomes of patients before (5 months, pre-intervention arm) and after (5 months, intervention arm) the implementation of SDD (Oral colistin syrup).

**Results:**

A total of 78 patients were included in the interventional arm with a mean age of 58.7 years whereas the pre-interventional group consisted of 94 study participants with a median age of 57.5 years. 11 out of 94 had positive respiratory sample culture (11.7%) in the preintervention group which mandated antibiotic therapy for HAP compared to one culture positive in the interventional period (OR 0.0980, 95% CI: 0.0124 to 0.777 and P=0.0279). One patient had blood stream infection in the pre-intervention period compared to none in the intervention phase. All-cause mortality in the pre-interventional group was 7.44% (7 in 94) vs 1.28% (1 in 78) in the interventional group (OR 0.1614, 95% CI: 0.0194 to 1.3416, P= 0.0914). Adverse events (nausea, vomiting & loose stools) were observed in a total of 24 study patients, but necessitated withdrawal of regimen only in nine patients.

**Conclusion:**

An SDD regimen of Colistin alone in Cardiac Surgery patients resulted in statistically significant reduction in incidence of Hospital Acquired Pneumonia, along with a reduction in all-cause mortality (though not statistically significant).

**Disclosures:**

**All Authors**: No reported disclosures

